# *p*-Aminobenzoate Organic Salts as Potential Plant Growth Regulators for Tomatoes

**DOI:** 10.3390/molecules25071635

**Published:** 2020-04-02

**Authors:** Radu-Liviu Sumalan, Lilia Croitor, Mihaela Petric, Isidora Radulov, Paulina Bourosh, Renata-Maria Sumalan, Manuela Crisan

**Affiliations:** 1 Faculty of Horticulture and Forestry, Banat′s University of Agriculture Science and Veterinary Medicine “King Michael Ist of Romania” from Timisoara, Calea Aradului nr 119, 300645, Timisoara, Romania; sumalanagro@yahoo.com (R.-L.S.); isidoraradulov@yahoo.com (I.R.); srenata_maria@yahoo.com (R.-M.S.); 2 “Coriolan Dragulescu” Institute of Chemistry, 24 Mihai Viteazul Blvd., 300223, Timisoara, Romania; croitor.lilia@gmail.com (L.C.); mihaelapetric@yahoo.com (M.P.); 3 Institute of Applied Physics, Academiei Street 5, MD2028, Chisinau, Moldova; bourosh.xray@phys.asm.md

**Keywords:** *p*-aminobenzoic acid, alkanolammonium salt, biological activity, plant growth regulator, crystal structure, supramolecular assembly

## Abstract

The discovery of environmentally friendly and inexpensive plant growth regulators (PGRs) for agronomically important crops is a necessity and must be considered a priority worldwide. This study provides the synthesis, structure determination and the biological evaluation of two binary organic salts as potential PGRs. New compounds have dual biological activity and are based on natural metabolite *p*-aminobenzoic acid (*p*ABAH) and different alkanolamines. Studied compounds exhibit hydrogen-bonded 3D supramolecular architectures with different crystal packing due to the formation of one homosynthon and various heterosynthons. The biological profile of new compounds was investigated in laboratory and greenhouse on *Solanum lycopersicum* L., revealing the efficiency in promoting plant rooting and plant productivity. The results may have a positive impact on agricultural economics, developing new sustainable PGRs for tomatoes.

## 1. Introduction

Growing populations have imposed the use of plant growth regulators (PGRs), which have become an integral part of agricultural and horticultural practices, maximizing crop production. To mitigate the harmful effects of conventional synthetic PGRs, such as 2,4-dichlorophenoxyacetic acid-2,4-D, on the environment and plant health [1], extensive efforts have been devoted to discovering sustainable alternatives. Since natural compounds extracted from plants or produced by bacteria rapidly degrade, limiting the applicability of the product [2], the use of PGRs based on synthetic analogues of natural products or active pharmaceutical ingredients commercially available are considered to be more effective [3]. Therefore, developing innovative, environmentally friendly and cost competitive PGRs remains an important task for researchers and a necessity at the global level for nutrition security. Understanding of chemical and crystal structure, inter- and intramolecular interactions as well as the structure–biological activity relationship is essential for investigation of new PGRs. Extensive studies show the importance of the carboxyl group and the planar aromatic ring in the structure of auxin-like PGRs, essential molecules that control almost every aspect of dormancy, seed germination and plant development [4].

Thus, benzoic acids, well-known for their importance as building blocks in drug development, gained increasing attention in plant science, being involved in various physiological processes from the regulation of seed germination [5,6,7] to disease resistance and stress tolerance in plants [8,9]. Among these compounds, *p*-aminobenzoic acid (*p*ABAH) is a well-known natural metabolite present in plant and animal tissues, widely described in literature as a precursor of folic acid [10] and recently of coenzyme Q [11]. It possesses numerous biological activities in medicine such as antioxidant [12], antibacterial [13], antimutagenic [14], anticoagulant [15], fibrinolytic and immunomodulating agent [16], protective drug against UV-irradiation [17] and also in agriculture as a chemical inducer associated with thermotolerance [18] and pathogens resistance [19] in plants. Moreover, an examination of the Cambridge Structural Database (CSD) has confirmed the *p*ABAH versatility as a fundamental building block in the design of soluble forms of organic multicomponent crystals and coordination compounds with various supramolecular architectures [20,21]. Besides being frequently used as co-formers, such as aliphatic and heterocyclic amines, alkanolamines are very few despite their lower toxicity [22], and their use as intermediates for the production of active pharmaceutical and cosmetic ingredients.

Our previous studies show the huge potential of alkanolamine-substituted benzoic acid systems which can generate supramolecular architectures with different topologies (1D, 2D and 3D) guided by different non-covalent interactions [23,24,25], have low toxicity [25,26], thermal stability [27] and promising applicability as auxin-like PGRs on the model plant *Arabidopsis thaliana* Col 0 [6]. The aim of this work was to develop new alkanolammonium *p*-aminobenzoates with characteristics of ideal PGRs: low toxicity, easily synthesized in laboratories, non-expensive, soluble in water and to investigate their role in growth and development of the most important vegetable cultivated in Romania and European Union in the last years, *Solanum lycopersicum* L. The discovery of new PGRs for commercial vegetables is essential, because vegetables play a vital role in food front, being the cheapest natural sources. Herein, two new alkanolammonium *p*-aminobenzoates were synthesized, structurally and physicochemically characterized and evaluated for their PGRs’ activity on tomatoes (*S. lycopersicum* L).

## 2. Results and Discussion

The new compounds were prepared by proton exchange reaction of *p*ABAH with different alkanolamines (HEEA-*p*ABA—ethylethanolammonium *p*-aminobenzoate; HDEEA-*p*ABA—diethylethanolammonium *p*-aminobenzoate) and isolated in excellent yields (>95%). The pKa rule [28] predicted the salts formation, because ΔpKa values pKa(alkanolamine)-pKa(*p*ABAH) were greater than three. Physicochemical and structural characterizations of new compounds are presented below.

### 2.1. Crystallographic Study

The single crystal X-ray diffraction data indicated that HEEA-*p*ABA crystallizes in the monoclinic centrosymmetric *P*2**_1_**/*c* space group, while HDEEA-*p*ABA crystallizes in the orthorhombic asymmetric *Pna*2**_1_** space group (Table 1).

**Figure 1:**
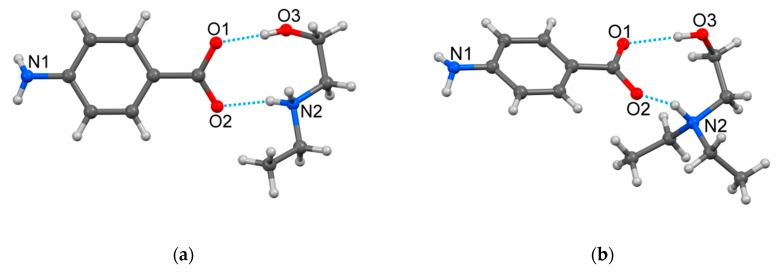
View of the HEEA-*p*ABA (**a**) and the HDEEA-*p*ABA (**b**) with partial atomic labelling and charge-assisted hydrogen bonds.

The analysis of both crystal structures revealed the formation of 1:1 organic salts HEEA-*p*ABA and HDEEA-*p*ABA with proton transfer from carboxylic group of *p*ABAH to nitrogen atoms of alkanolamine molecules (Figure 1). The (*p*ABA)^‒^ anion formed non-planar system in HEEA-*p*ABA and a practically planar system in HDEEA-*p*ABA, since the dihedral angle between the least squares plane of the phenyl ring C_6_ and the COO^‒^ group was equal to 20.1° and 5.6°, respectively.

The nitrogen atoms in the amino group in both structures had almost a pyramidal configuration, its valence angles being equal to 108.07, 109.00 and 111.22° in (HEEA)^+^ of HEEA-*p*ABA, and 108.80, 109.83 and 110.01° in (HDEEA)^+^ of HDEEA-*p*ABA. The (HEEA)^+^ and (HDEEA)^+^ cations adopt the Syn-Clinal conformation with the N(2)CCO(3) torsion angles equal to −66.90 and −66.65°, respectively. The alkanolamine cations and (*p*ABA)^‒^ anions were held together by two charge-assisted O‒H∙∙∙O^‒^ and N^+^‒H∙∙∙O hydrogen bonds in HEEA-*p*ABA as distances C‒O(1) and C‒O(2) are equal to 1.270(2) and 1.254(3) Å and one charge-assisted N^+^‒H∙∙∙O^‒^ and one classic O‒H∙∙∙O hydrogen bonds in HDEEA-*p*ABA with C‒O(1) and C‒O(2) distances equal 1.254(8) and 1.276(8) Å. As a result, in both compounds, a similar *R*_2_^2^(9) graph set was formed. In both structures, the NH_2_ group of (*p*ABA)^‒^ anion participated to charge-assisted N‒H∙∙∙O^‒^ and classic N‒H∙∙∙O hydrogen bonds with carboxylic oxygen atoms of adjacent *p*ABA anions (Table 2).

**Figure 2:**
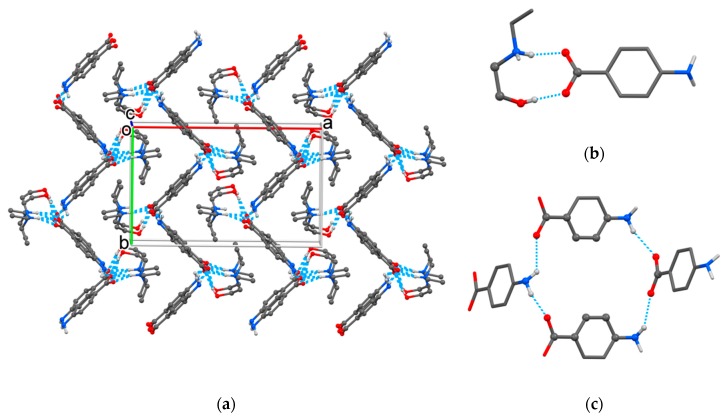
The crystal packing of HEEA-*p*ABA (**a**) with representation of *R*_2_^2^(9) heterosynthon (**b**) and *R*_4_^4^(22) homosynthon in crystal (**c**).

Cations and anions in the HEEA-*p*ABA structure were held together in a supramolecular network (Figure 2a) obtained from *R*_2_^2^(9) heterosynthons (Figure 2b) and *R*_4_^4^(22) homosynthons formed by four anions (Figure 2c) with the involvement of fine C‒H∙∙∙O hydrogen bond between amine and carboxylic groups (Table 2), in addition to the mentioned N(O)‒H∙∙∙O hydrogen interaction.

**Figure 3:**
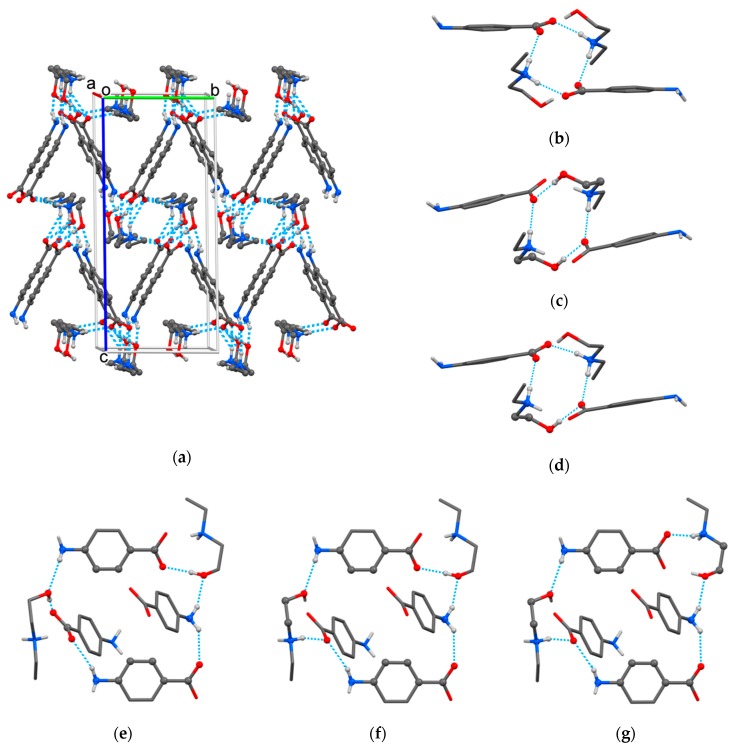
The crystal packing (**a**) and multicomponent heterosynthons found in HDEEA-*p*ABA crystal: *R*_4_^4^(12) (**b**), *R*_2_^4^(14) (**c**), *R*_3_^4^(13) (**d**), *R*_6_^6^(26) (**e**), *R*_5_^6^(27) (**f**) and *R*_5_^6^(30) (**g**).

The crystal packing of HDEEA-*p*ABA (Figure 3a) was based on seven heterosynthons between HDEEA cations and *p*ABA anions: bicomponent *R*_2_^2^(9) heterosynthon discussed above, three tetracomponent heterosynthons formed by two cations and two anions (Figure 3b–d) and three hexacomponent heterosynthons assembled by two cations and four anions (Figure 3e–g). The crystalline structure was additionally stabilized by fine C‒H∙∙∙O hydrogen bonds with the involvement of C(8) and C(13) as donors of H (Table 2).

### 2.2. FTIR Spectroscopy

**Figure 4:**
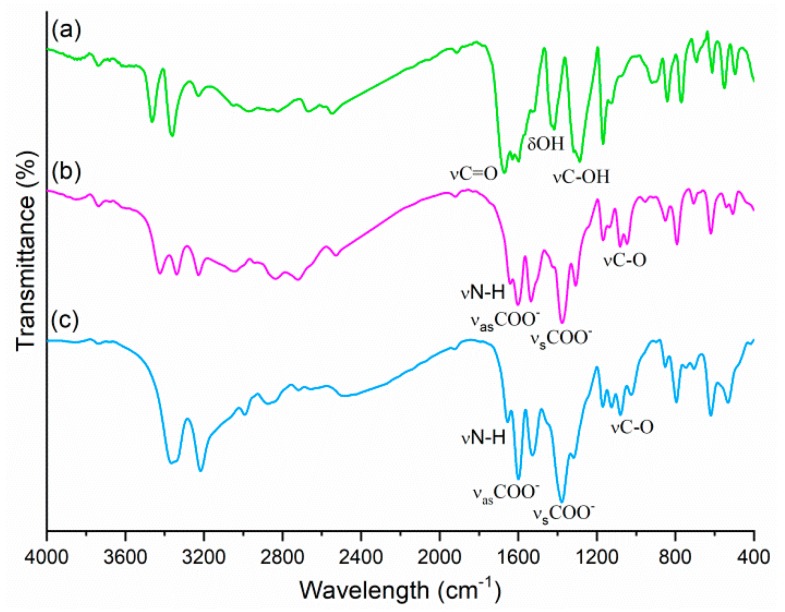
FTIR spectra of *p*ABAH (**a**), HEEA-*p*ABA (**b**) and HDEEA-*p*ABA (**c**).

The FTIR spectroscopic investigations (Figure 4) provide a supplementary insight into alkanolammonium salts formation by exhibiting the differences in the intensities and wavenumbers of C=O, N‒H and O‒H stretching modes in new compounds compared to the corresponding acid.

The HEEA-*p*ABA and HDEEA-*p*ABA spectra had certain similarities: the presence of strong bands responsible for the symmetric (1378 cm^‒1^; 1381 cm^‒1^) and asymmetric (1602 cm^‒1^; 1599 cm^‒1^) stretching vibrations of carboxylate group which do not exist in the spectra of *p*ABAH, as well as the absence of νC=O (1671 cm^‒1^), νC‒OH (1287 cm^‒1^) and δOH (1417 cm^‒1^) bands characteristic of the carboxylic group [29]. The lower values for symmetric and asymmetric νCOO^‒^ stretching vibrations compared to the free acid indicate that carbonyl was H-bonded in the anion–cation and anion–anion systems. Furthermore, the absence of bands characteristic of the –COOH group confirmed the formation of HEEA-*p*ABA and HDEEA-*p*ABA salts.

The strong and narrow absorption bands (3464 cm^‒1^ and 3360 cm^‒1^) assigned for asymmetric and symmetric NH_2_ stretching vibrations in *p*ABAH spectrum decreased in intensity, and wavenumbers in the case of two alkanolammonium salts, becoming 3425 cm^‒1^ and 3341 cm^‒1^ for HEEA-*p*ABA and 3367 cm^‒1^ for HDEEA-*p*ABA. These indicate N‒H∙∙∙O^‒^ and N‒H∙∙∙O intermolecular interactions between anion–anion in HEEA-*p*ABA and anion–anion/anion–cation in HDEEA-*p*ABA. The broad bands in the range 3200–2700 cm^‒1^ are attributed to O‒H stretching vibrations and overlap with NH_2_^+^ vibration band (3043 cm^‒1^) in the case of secondary alkanolamine. Weak bands of N‒H deformation vibrations were observed at 1640 cm^‒1^ and 1655 cm^‒1^, respectively, in salt spectra. Supplementary proof of the salt formation was confirmed by the appearance in HEEA-*p*ABA and HDEEA-*p*ABA spectra of C‒O stretching vibrations at 1100–1000 cm^−1^, belonging to alkanolamines. The differences between two new alkanolammonium salts spectra also illustrate the diversity of the synthons in crystals.

### 2.3. Thermal Analysis

**Figure 5:**
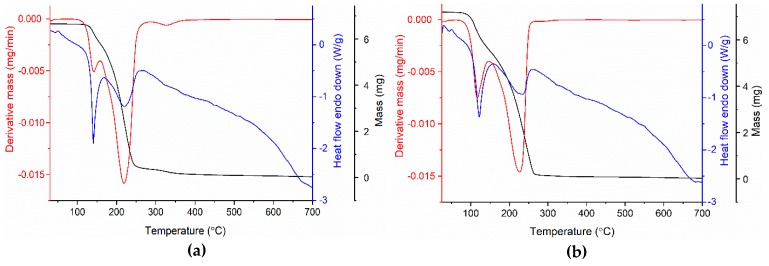
Thermo-analytical curves of HEEA-*p*ABA (**a**) and HDEEA-*p*ABA (**b**).

The salts were characterized by thermal analysis (thermogravimetric analysis—TGA; derivative thermogravimetry—DTG; and heat flow—HF) in order to determine thermal stability (Figure 5). The TGA and DTG curves show that HEEA-*p*ABA and HDEEA-*p*ABA are thermally stable up to 124 °C and 90 °C, respectively when they begin to decompose into two steps with a total mass loss of over 99.4%.

Therefore, HEEA-*p*ABA had a first mass loss of 13.1% in the range 124.77 °C–160.15 °C, probably due to the removal of the ethyl group from the cation. This decomposition process was accompanied by melting at 138.52 °C. The second step up to 700 °C corresponded to 81.68% mass loss and complete decomposition of the compound. In the case of HDEEA-*p*ABA, the decomposition process, up to 700 °C, began with a 22.59% mass loss due to the elimination of diethyl groups from the cation and continued with the second step which involved a final 76.25% mass loss ascribed to the C_9_H_12_N_2_O_3_ molecule. The DTG curve accompanied the decomposition process by one sharp peak at 115.87 °C and another broad peak at 226.13 °C. HF curve showed a first strong and sharp endothermic peak at 120.85 °C, attributed to the melting point of the compound. Thermogravimetric studies indicate that the studied compounds were anhydrous and HEEA-*p*ABA was more thermally stable than HDEEA-*p*ABA.

### 2.4. Biological Studies

The auxin-like PGRs’ effect on new compounds was investigated in laboratory and greenhouse conditions, on tomato, the main commercial vegetable cultivated in Romania and Europe in the last years. Commercial indole-3-acetic acid (IAA), the most abundant and studied natural auxin in plants, was used as the reference compound. The results of experiment 1 (Table 3) showed that new compounds (HEEA-*p*ABA, HDEEA-*p*ABA) and IAA inhibited germination at all tested concentrations or even blocking it (e.g., IAA, at concentrations above 0.5 mM) especially in the first interval of treatment (after 7 days), compared with the control. The results obtained show that increasing the concentration leads to inhibition of germination. Thus, in the case of HEEA-*p*ABA application, increasing concentration from 0.1 mM to 0.5 mM and then to 1 mM, reduced significantly seed germination by about 20% and 9%, respectively. Instead, HDEEA-*p*ABA decreased seed germination by approximately 24.5% only from 0.5 mM to 1 mM.

A tendency towards uniformization of values was revealed ten days after the induction of germination. An increase of germination with approximately 22% was observed only at the concentration of 0.1 mM, for HDEEA-*p*ABA treatment compared to IAA. In the case of 0.5 and 1 mM concentrations, HEEA-*p*ABA and HDEEA-*p*ABA treatments had similar effects; the increase of the concentration did not significantly influence seed germination. Analysis of the effects of the three treatments at the same concentration level shows that the treatments with HEEA-*p*ABA and HDEEA-*p*ABA had significantly higher effects than IAA on tomato seed germination.

Recent studies have characterized auxins as regulators that act positively by maintaining dormancy [30] and negatively by inhibiting seed germination [31]. The hormonal signalling of natural auxin IAA has often been associated with physiological processes manifested during plant growth and development, including seed germination [32]. Some previous studies have shown that exogenous application of IAA inhibits and delays seed germination [33,34]. The mechanism underlying germination blockade is not yet fully elucidated, but it seems to be due to the fact that auxin induces seminal break by enhancing signal transduction of abscisic acid, which associates auxin with maintaining seminal dormancy [30].

Therefore, the results of the tested compounds on tomato seeds germination confirm that IAA shows clear germination blocking effects, and the new compounds, even if they belong to the same category of compounds, have inhibitory effects only in the first part of the interval, the differences from the control being insignificant after a 10 day treatment.

Otherwise there are some studies that prove that besides phytohormones, there are chemical compounds that have the ability to regulate plant growth and development, such as nitrogen oxide and ROS (superoxide; hydrogen peroxide; hydroxyl radical; hydroxyl ion; and nitric oxide), both types being proven to regulate seed dormancy and germination [35,36,37,38].

Regarding experiment 2 (Table 4), the chlorophyll content of tomato seedlings was influenced by HEEA-*p*ABA and HDEEA-*p*ABA treatments, showing a significant increase compared to the control and IAA. The effect of new treatments on morphological characters in tomato seedlings indicated that plant height and primary root length presented variations comparatively with control and IAA, while maximum length of secondary roots had similar values to control and significantly smaller effect to IAA. The number of secondary roots (SR) registered the highest values under the effect of IAA treatment compared to the other treatments. The HEEA and HDEEA generated a significant increase (more than 86% of the number of SR) compared to the control.

It is known that auxin plays an important role in controlling the roots growth and plant development. The total amount of this major phytohormone is generated by the local biosynthesis and transport. The natural auxin IAA reveals an inhibitory effect on primary root (PR) growth. SR are essential components of the root system which contribute to maximizing the absorption capacity of water and nutrients, facilitating rapid adaptation to environmental changes [39,40]. It is well known that IAA is involved in each stage of SR′s formation, the disorders in auxin biosynthesis and transport resulting in a reduced number of SR [41,42,43,44,45].

Therefore, our results attest that HEEA-*p*ABA and HDEEA-*p*ABA treatment solutions stimulate the plants height and the length growth of PR in contrast to IAA, which has inhibitory effects. Also, new compounds promote the formation of a large number of SR and support their growth, compared to control. Contrary, exogenous IAA generates the formation of a large number of SR (104.5 ± 18.42), probably due to the ability of acropetal migration within the root [46], but without ensuring their length growth. These results can also be correlated with the higher capacity of chlorophyll biosynthesis in variants treated with 0.5mM HEEA-*p*ABA and HDEEA-*p*ABA exogenous solutions.

The dynamics of the plants height (Table 5) shows that the foliar treatments with HDEEA-*p*ABA and IAA have a similar and significantly superior influences to the treatments with HEEA-*p*ABA and control, in all the stages of development, from the beginning of flowering until the first stage of fruit emergence.

The three treatments and control did not differ significantly in their effect on the number of leaves at the beginning of flowering (Table 6). Significantly higher number of leaves compared to control has been stimulated by IAA during the flowering period and by HDEEA-*p*ABA treatment at the emergence of first fruiting floor of tomato plants. All treatments presented a significant increase in the number of leaves from the beginning of flowering to full flowering, with differences between 3.33 in HEEA-*p*ABA and 5.33 in HDEEA-*p*ABA. In the period from flowering to the emergence of first fruiting floor, the number of leaves per plant showed little and no significant variations in all cases.

It is known that auxins can stimulate plant growth by enhancing photosynthesis due to the fact of chlorophyll increases [47]. The chlorophyll pigments are probably the most relevant natural biomolecules due to the fact of their importance in photosynthesis. Therefore, a direct correlation between their quantity and gross primary productivity has been demonstrated [48]. Our results present a significant increase in chlorophyll content induced by HDEEA-*p*ABA (41.43 ± 0.69) and a significantly lower one for HEEA-*p*ABA (37.07 ± 1.74) compared to IAA treatment and control, at the beginning of flowering (Table 7). In the flowering and emergence of first fruiting floor periods, the highest chlorophyll content was determined in the tomato plants treated with HDEEA-*p*ABA and IAA, respectively, while in the variants HEEA-*p*ABA and control, the chlorophyll content was lower. Control plants recorded a significant increase of chlorophyll content from the beginning of flowering to the emergence of first fruiting floor, in contrast to treated plants, which showed a notable increase only between the first two phenophases.

These results support the hypothesis that the initiation of the flowering and fruiting processes, which means the passage of plants from the vegetative to the generative stage, diminishes or stagnates the dynamics of chlorophyll biosynthesis, the plant concentrating the metabolic activity towards the biosynthesis of photosynthetic compounds such as carbohydrates. Therefore, the cellular metabolism was modified by reducing the biosynthesis of functional molecules, with nitrogen, and stimulating the synthesis of structural biomolecules, with carbon [49].

There are studies which show that foliar application of auxinic compounds, at different concentrations, induced increases in plant height, fresh and dried mass, number of shoots and leaves per plant, as well as productivity-related components [50,51]. Other research, on the contrary, concluded that the application of low or moderate doses of exogenous auxins did not generate significant changes on the plant growth parameters and the high doses even had effects of reducing the values of these parameters compared to the untreated variants [52,53,54,55].

The foliar apparatus, or the number of leaves per plant, is the essential component of the photosynthetic process. The leaves are considered the primary photosynthetic organs, and their number, along with their surface, determines the amplitude of the assimilation process. Previous studies have shown that phytohormones, especially auxin (IAA) and gibberellins (GA), play a key role in the emergence and development of foliar apparatus [56]. The results prove the stimulatory effect of foliar auxin application in tomatoes plant growth, the best results being obtained by using HDEEA-*p*ABA and IAA, respectively, at a 0.5 mM concentration. Plants with a large number of leaves show an increased photosynthetic potential, which allows the synthesis, transport, and accumulation of larger amounts of valuable bioactive compounds [57,58].

## 3. Materials and Methods

### 3.1. Materials and Physical Measurements

Reagents *p*ABAH, EEA (ethylethanolamine) and DEEA (diethylethanolamine) were purchased from Fluka Chemie AG (Buchs SG, Switzerland)) in analytical purity and were used without further purification. Melting points of recrystallized compounds were determined on a Boetius melting point apparatus and were uncorrected. The FTIR spectra (KBr pellet) were recorded on a JASCO-FTIR-4200 spectrometer (Easton, MD, USA), in the range 4000–400 cm^−1^ with a resolution of 4.0 cm^−1^ and a scanning speed of 16 mm s^−1^. Thermal analysis was performed using a TGA/SDTA 851-LF 1100 Mettler apparatus (Columbus, OH, USA) The sample weight of about 7 mg was used for the test. The measurements were carried out in a dynamic air atmosphere, in the temperature range of 25–700 °C with a heating rate of 10 °C min^−1^.

### 3.2. Synthesis Procedure of HEEA-pABA and HDEEA-pABA

New compounds were prepared in a 1:1 molar ratio, adding dropwise EEA (0.71 mL, 7.28 mmol) and DEEA (0.97 mL, 7.3 mmol) respectively into a solution of *p*ABA (1 g, 7.29 mmol) in acetone (20 mL), under constant stirring at room temperature. White microcrystalline precipitates in high yields (>95 %) were formed after the addition of alkanolamines. The compounds were collected by filtration, washed with acetone, and dried in air. Colourless crystals suitable for single-crystal X-ray diffraction analysis were obtained after few days by slow evaporation in acetone. Melting points (Boetius) were: HEEA-*p*ABA m.p. = 140–141 °C and HDEEA-*p*ABA m.p. = 122–123 °C.

### 3.3. Single Crystal X-ray Diffraction Study

Diffraction measurements for compounds HEEA-*p*ABA and HDEEA-*p*ABA were carried out at room temperature on an Xcalibur E diffractometer (Abingdon, Oxfordshire, United Kingdom) equipped with a CCD area detector and a graphite monochromator utilizing MoKα radiation. Final unit cell dimensions were obtained and refined on an entire data set. All calculations to solve the structures and to refine the proposed models were carried out with the SHELXL2014 program package [59]. All non-hydrogen atoms were refined anisotropically. Hydrogen atoms attached to carbon, nitrogen and oxygen atoms were positioned geometrically and treated as riding atoms. The X–ray data and the details of the refinement for both compounds are summarized in Table 1, and selected geometric parameters are given in Table 2. Figure 1, Figure 2 and Figure 3 were produced using the Mercury program (Cambridge, United Kingdom) [60]. Crystallographic data of the new compounds reported herein were deposited with the Cambridge Crystallographic Data Centre (Cambridge, United Kingdom) and allocated under the deposition numbers CCDC 1989303–1989304 (Supplementary Materials).

### 3.4. Biological Assays

Seeds of tomato cultivar named Tomtim created by BUASVM Timisoara, included in the CPVO (Community Plant Variety Office) test protocols, UPOV (The International Union for the Protection of New Varieties of Plants) and National Tests Guidelines were used in this study.

#### 3.4.1. Germination Tests (Experiment 1)

Tomatoes’ mature seeds were surface sterilized by keeping them in a commercial 50% (v/v) sodium hypochlorite solution for 10 min and rinsed three times with distilled water before transferring them to Petri dishes. Twenty-five sterilized seeds were each placed into a 9 cm Petri dish containing two Whatman filter papers moistened with 5 mL solution of MES (2-(N-morpholino)ethanesulfonic acid) 5 mM and CaSO4 0.5 mM (pH 6) for control and different concentrations (0.1, 0.5 and 1 mM) of HEEA-*p*ABA, HDEEA-*p*ABA or IAA solutions for treated samples. Each experiment was repeated three times. Petri dishes of all variants (control and treated) were placed in a growth chamber at 25 ± 2 °C, 70%–75% relative humidity, and 16/8 light/dark conditions. The seeds with emerged radicle were counted at 7 and 10 days and percentage were calculated.

#### 3.4.2. Seedling Growth Tests (Experiment 2)

Germinated seeds of each control and 0.5 mM treated samples were transplanted into pots (200 mL) with peat. Two germinated seeds/pot were placed in 15 replications for each experimental variant as follows: control watered with distilled water and other variants treated with 0.5 mM IAA, HEEA-*p*ABA and HDEEA-*p*ABA solutions. The treatments were performed by applying the solutions in pots, at roots, 25 mL at intervals of 7 days. The determinations were made after 3 applications, after 21 days of growing plants in the greenhouse (25/18 °C, day/night; 65% rh, and 12/12 light/dark conditions). Parameters included seedling height (cm), chlorophyll content (SPAD), main root length (cm), number and maximum length of secondary roots (cm) were determined.

#### 3.4.3. Tests on Tomato Plants (Experiment 3)

After 56 days, 10 seedlings from each experimental variant were planted in the greenhouse, on a fertile soil substrate with fermented manure (5 kg/sqm). The same experimental variants were maintained as in the case of seedling tests, being carried out three treatments with foliar application, in doses correlated with the plant sizes of 8 mL/plant (the first treatment), 12 mL/plant (2nd) and 16 mL/plant (third) at 7 day intervals.

#### 3.4.4. Data and Statistical Analysis

In this study, ten replicates of each moment of measurement were carried out for each sample, regarding the effect of different plant growth regulator substances and concentrations. All data were expressed as means ± standard error of the mean (*SEM*). Means were compared using least significant difference (*LSD*) test. The significant differences between the sample means (*p* < 0.05) were expressed by different letters.

## 4. Conclusions

Two new binary organic salts of *p*ABA with different alkanolamines were successfully synthesized and characterized, and their auxin-like PGRs’ properties investigated in laboratory and greenhouse on *Solanum lycopersicum* L. by comparison with the most abundant and studied natural auxin in plants, IAA. The single crystal structures’ interpretation was correlated with the FTIR spectral data showing the intermolecular interactions established within the salts. Both new structures exhibited hydrogen-bonded supramolecular network architectures obtained from: (i) *R*_2_^2^(9) heterosynthons and *R*_4_^4^(22) homosynthons in HEEA-*p*ABA; and (ii) *R*_2_^2^(9), *R*_4_^4^(12), *R*_2_^4^(14) and *R*_3_^4^(13), *R*_6_^6^(26), *R*_5_^6^(27) and *R*_5_^6^(30) heterosynthons in HDEEA-*p*ABA. The presence of *R*_2_^2^(9) graph set in both structures indicates the relevance of charge-assisted N^+^‒H∙∙∙O^‒^ hydrogen bond in the formation of salts. The results obtained by investigating the biological profile of new alkanolammonium *p*-aminobenzoates as potential PGRs reveal the possibility of their use in stimulating the rooting and growth processes of tomato seedlings, as well as the positive involvement in plant growth and chlorophyll pigment biosynthesis. Considering the distinct supramolecular synthons in the design of molecular crystals and the promising biological results, this study sheds light on the alkanolammonium *p*-aminobenzoates as potential alternatives in the search of sustainable PGRs for tomatoes.

## Figures and Tables

**Table 1:** Crystallographic data and structure refinement details for new compounds.

Compound	HEEA-*p*ABA	HDEEA-*p*ABA
Empirical formula	C_11_H_18_N_2_O_3_	C_13_H_22_N_2_O_3_
*Fw*	226.27	254.32
*T* (K)	293(2)	293(2)
Crystal system	Monoclinic	Orthorhombic
Space group	*P*2_1_/*c*	*Pna*2_1_
*a* (Å)	8.3465(4)	17.9492(17)
*b* (Å)	8.1802(5)	11.1812(9)
*c* (Å)	18.8676(9)	7.1445(6)
β (°)	102.684(5)	90
*V* (Å^3^)	1256.76(11)	1433.9(2)
*Z*	4	4
ρ_calcd_ (g cm^−3^)	1.196	1.178
μ (mm^−1^)	0.087	0.084
Crystal size (mm)	0.60 × 0.25 × 0.05	0.400 × 0.200 × 0.040
2Θ range (°)	3.332 to 24.990	3.569 to 24.996
Reflections collected/unique	4344/2199 (*R_int_* = 0.0243)	3177/1870 (*R_int_* = 0.0417)
Reflections with (*I* > 2 δ(*I*))	1576	1059
Parameters	148	167
*R*_1_, w*R*_2_ (*I* > 2 δ(*I*))	0.0528, 0.1382	0.0609, 0.1259
*R*_1_, w*R*_2_ (all data)	0.0774, 0.1564	0.1147, 0.1484
GOF ^[c]^	1.000	1.000
Largest diff. peak/hole (e Å^−^^3^)	0.332/−0.306	0.178/−0.151

**Table 2:** Hydrogen bond distances (Å) and angles (°) in HEEA-*p*ABA and HDEEA-*p*ABA.

D‒H∙∙∙A	d(D–H)	d(H∙∙∙A)	d(D∙∙∙A)	∠(DHA)	Symmetry Transformations for Acceptor
**HEEA-*p*ABA**					
O(3)‒H(1)∙∙∙O(1)	0.82	1.83	2.650(2)	178	*x*, *y*, *z*
N(1)‒H(1)∙∙∙O(3)	0.87	2.27	3.077(3)	155	−*x* + 1, *y* + 1/2, −*z* + 1/2
N(1)‒H(2)∙∙∙O(2)	0.87	2.34	3.010(3)	135	−*x*, *y* + 1/2, −*z* + 1/2
N(2)‒H(1)∙∙∙O(1)	0.89	1.89	2.775(2)	174	−*x* + 1, −*y* + 1, −*z* + 1
N(2)‒H(2)∙∙∙O(2)	0.89	1.92	2.787(2)	165	*x*, *y*, *z*
C(8)‒H(8)∙∙∙O(3)	0.97	2.62	3.508(3)	152	−*x* + 1, −*y*, −*z* + 1
**HDEEA-*p*ABA**					
O(3)‒H(3)∙∙∙O(1)	0.82	1.87	2.676(5)	170	*x*, *y*, *z*
N(1)‒H(1)∙∙∙O(2)	0.87	2.09	2.908(8)	158	−*x* + 3/2, *y* + 1/2, *z* + 1/2
N(1)‒H(2)∙∙∙O(1)	0.87	2.29	2.858(9)	123	−*x* + 3/2, *y* + 1/2, *z*−1/2
N(2)‒H(2)∙∙∙O(2)	0.98	1.78	2.701(6)	156	*x*, *y*, *z*
C(8)‒H(8)∙∙∙O(3)	0.97	2.54	3.501(9)	172	−*x* + 1, −*y* + 1, *z*−1/2
C(13)‒H(8)∙∙∙O(2)	0.96	2.60	3.285(8)	129	*x*, *y*, *z*

**Table 3:** The influence of treatments on tomato seed germination (tomato cultivar (cv.) Tomtim).

Moment	After 7 Days			After 10 Days		
Treatment	0.1 mM	0.5 mM	1 mM	0.1 mM	0.5 mM	1 mM
Control (MES)	91.11 ^a^	91.11 ^a^	91.11 ^a^	100 ^a^	100 ^a^	100 ^a^
Indole-3-acetic acid (IAA)	^x^ 8.89 ^c^	^x^ 0 ^d^	^x^ 0 ^c^	^x^ 75.56 ^b^	^y^ 0 ^b^	^y^ 0 ^b^
HEEA-*p*ABA	^x^ 46.67 ^b^	^y^ 26.67 ^c^	^y^ 17.78 ^b^	^x^ 88.89 ^ab^	^x^ 93.33 ^a^	^x^ 91.11 ^a^
HDEEA-*p*ABA	^x^ 40.00 ^b^	^x^ 44.44 ^b^	^y^ 20.00 ^b^	^x^ 97.78 ^a^	^x^ 93.33 ^a^	^x^ 88.89 ^a^
Treatment. *LSD_5%_* = 17.68 (a,b,c,d); Concentration. *LSD_5%_* = 11.21 (x,y)				Treatment. *LSD_5%_* = 16.64 (a,b); Concentration. *LSD_5%_* = 17.11 (x,y)		

The differences among the variants noted with different letters are considered significant (*p* < 0.05).

**Table 4:** The influence of treatments on tomato seedlings (cv. Tomtim).

Character/ Treatment	Height (cm)	Chlorophyll (SPAD)	Primary Root Length (cm)	No of Secondary Roots	Maximum Length of Secondary Roots (cm)
Control (MES)	5.50±0.50 ^b^	31.20 ± 1.49 ^c^	11.10 ± 0.37 ^ab^	25.00 ± 2.99 ^c^	9.27 ± 0.32 ^a^
IAA	3.20 ± 0.18 ^c^	32.25 ± 0.25 ^c^	8.65 ± 0.55 ^c^	104.50 ± 18.42 ^a^	3.50 ± 0.55 ^b^
HEEA-*p*ABA	5.25 ± 0.75 ^b^	38.20 ± 0.60 ^a^	11.50 ± 0.50 ^a^	46.50 ± 8.47 ^b^	8.28 ± 0.43 ^a^
HDEEA-*p*ABA	6.25 ± 0.95 ^a^	36.15 ± 0.25 ^b^	10.50 ± 0.50 ^b^	51.00 ± 1.00 ^b^	8.39 ± 0.37 ^a^
*LSD*_5%_	0.61	1.51	0.59	15.93	1.28

The differences among the variants noted with different letters are considered significant (*p* < 0.05).

**Table 5:** The treatments effect on height (cm) of tomato plants (cv. Tomtim) in different stages of development.

Development Stage/ Treatments	Beginning of Flowering	Flowering	Emergence of First Fruiting Floor
Control (MES)	^z^ 42.50 ± 3.07 ^b^	^z^ 66.75 ± 5.02 ^b^	^yz^ 83.75 ± 7.53 ^b^
IAA	^z^ 49.00 ± 0.58 ^a^	^z^ 77.00 ± 2.89 ^a^	^yz^ 96.67 ± 3.67 ^a^
HEEA-*p*ABA	^z^ 43.00 ± 4.51 ^b^	^z^ 64.67 ± 4.67 ^b^	^yz^ 85.67 ± 7.85 ^b^
HDEEA-*p*ABA	^x^ 47.00 ± 1.00 ^a^	^zx^ 79.67 ± 1.20 ^a^	^yz^ 104.33 ± 3.39 ^a^

Treatments *LSD_5%_* = 4,36 (a,b); developmental stage *LSD_5%_* = 48.87 (x,y,z). The differences among the variants noted with different letters are considered significant (*p* < 0.05).

**Table 6:** The influence of treatments on leaves number of tomato plants (cv. Tomtim) in different stages of development.

**Development Stage/ Treatments**	**Beginning of Flowering**	**Flowering**	**Emergence of First Fruiting Floor**
Control (MES)	^y^ 6.50 ± 0.50 ^a^	^x^ 10.50 ± 0.87 ^b^	^x^ 12.75 ± 0.48 ^b^
IAA	^y^ 7.33 ± 0.67 ^a^	^x^ 12.33 ± 0.67 ^a^	^x^ 13.67 ± 0.88 ^ab^
HEEA-*p*ABA	^y^ 7.33 ± 0.33 ^a^	^x^ 10.67 ± 1.45 ^b^	^x^ 13.33 ± 0.88 ^ab^
HDEEA-*p*ABA	^y^ 6.67 ± 0.88 ^a^	^x^ 12.00 ± 1.00 ^ab^	^x^ 14.33 ± 0.67 ^a^

Treatments *LSD5%* = 1.54 (a,b); Developmental stage *LSD5%* = 3.24 (x,y). The differences among the variants noted with different letters are considered significant (*p* < 0.05).

**Table 7:** Effect of treatments on chlorophyll content (SPAD) of tomato plants’ foliar apparatus (cv. Tomtim) in different stages of development.

Development Stage/ Treatments	Beginning of Flowering	Flowering	Emergence of First Fruiting Floor
Control (MES)	^u^ 39.13 ± 1.43 ^b^	^z^ 47.00 ± 2.14 ^b^	^yz^ 48.75 ± 0.70 ^b^
IAA	^y^ 39.33 ± 1.53 ^b^	^x^ 51.50 ± 1.45 ^a^	^x^ 50.73 ± 0.69 ^a^
HEEA-*p*ABA	^z^ 37.07 ± 1.74 ^c^	^y^ 47.43 ± 2.38 ^b^	^y^ 49.30 ± 1.46 ^b^
HDEEA-*p*ABA	^y^ 41.43 ± 0.69 ^a^	^x^ 50.37 ± 0.03 ^a^	^x^ 51.17 ± 0.23 ^a^

Treatments *LSD_5%_* = 0.64 (a,b,c); developmental stage *LSD_5%_* = 6.73 (u,x,y,z). The differences among the variants noted with different letters are considered significant (*p* < 0.05).

## Supplementary Materials

Cifs for title compounds are available online.
